# A review of root, tuber and banana crops in developing countries: past, present and future

**DOI:** 10.1111/ijfs.14778

**Published:** 2020-10-13

**Authors:** Gregory J. Scott

**Affiliations:** ^1^ CENTRUM‐PUCP Graduate Business School Jirón Daniel Alomía Robles 125, Santiago de Surco 15023 Lima 33 Perú; ^2^ Pontificia Universidad Católica del Perú Av. Universitaria 1801, San Miguel 15088 Lima 32 Perú

**Keywords:** Banana, cassava, potato, plantain, sweetpotato, yam, production, consumption, income, food security

## Abstract

For many of the developing world's poorest farmers and food‐insecure people, roots, tubers, bananas and plantain crops (RTBs) serve as a critical source of food, nutrition and cash income. RTBs have been particularly important in areas where local agri‐food systems are under stress. Under such circumstances, growers, processors and traders often see opportunities to improve food security or increase their incomes with those crops due to shifting tastes and preferences for food and non‐food products. Since the early 1990s, cassava output surged in sub‐Saharan Africa, while potato production expanded rapidly in Asia. RTBs are consumed by over three billion people in developing countries with a market value of US$ 339 billion. This paper analyses the major changes in production, utilisation and trade of RTBs over the last six decades, assesses estimates of their future trajectory and offers recommendations so that they might achieve their full potential.

## Graphical abstract

For many of the developing world's poorest farmers and food‐insecure people, roots, tubers, banana and plantain crops (RTBs) serve as a critical source of food, nutrition and cash income. Although sometimes seen as locked in traditional, unchanging agri‐food systems, trends in production for RTBs have been both dynamic and diverse over the last six decades as this figure illustrates. This paper analyses the driving factors behind the evolution in production, utilisation and trade of RTBs in developing countries since the early 1960s and recent estimates of their future trajectory.

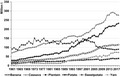

## Introduction

Many of the developing world's poorest farmers and food‐insecure people are dependent on roots, tubers, bananas and plantain crops (RTBs hereafter) as a contributing source of food, nutrition and cash income. RTBs have been particularly important in those regions or countries experiencing rapid population growth and where local agri‐food systems are under stress, for example due to droughts, spikes in commodity prices limiting capacity for food imports. Under such circumstances, growers, processors and traders often see RTBs as offering opportunities not only to improve household food security but also to increase their incomes due to shifting tastes and preferences for food and non‐food products (Alexandratos & Bruinsma, 2012; Nweke, 2016; Spencer & Ezedinma, 2017; Lescot, 2020; Kwa & Temple, 2019; Scott *et al*., 2019a; Low & Thiele, 2020). So much so that RTBs are currently consumed by over three billion people in developing countries and as calculated in greater detail below have an estimated annualized market value of US$ 339 billion. Given the importance of RTBs, continued population growth, massive urbanisation and growing concerns about future food supplies with the advent of climate change have raised questions about their future trajectory. The potential implications of that trajectory are of particular interest for growers, processors, traders and urban consumers of RTBs as well as policymakers and researchers.

This paper aims to provide a global, historical context for discussion and debate regarding the opportunities that RTBs offer to improve food supplies, nutrition and incomes for both urban poor and rural poor in developing countries. It utilises an agri‐food system framework to analyse past trends and future prospects, based on FAO time‐series data over the last six decades and a select review of previous publications. An agri‐food system approach focuses on production through to utilisation for a particular food commodity. In that regard, recent research on RTBs has tended to focus either on specific crops in specific regions or on all RTBs taken together at the global level. This paper combines both perspectives to provide a more consolidated assessment. In so doing, the paper does not pretend to offer an exhaustive review of all the topics and issues related to RTBs in developing countries, but rather focuses on the major factors that influenced production and use of RTBs in the past as well as their future trajectory.

After a brief review of production and use of RTBs as a group, the paper then analyses trends for each of the major ones. Coverage varies to reflect their respective importance. An aggregate review of RTBs as a group including their current estimated market value follows next. The paper concludes by noting the most recent projections for a selection of RTBs to 2030 and 2050 and some key factors influencing each going forward.

### Major global developments for RTBs

**Figure 1:**
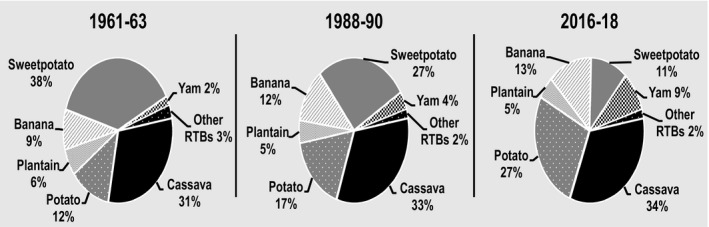
Percentages of the sum of total production of cassava, potato, sweetpotato, banana, yam, plantain and other RTBs^a^ produced in developing countries in selected years, 1961–2018.^b a^Others here refers to taro, yautia and the FAO category 'R&T, nes' that includes arracacha, mashua, ullucu, yacon, and other root and tuber crops. ^b^FAOSTAT statistics for RTBS produced in developing countries in Africa, Asia and Latin America and the Caribbean (LAC) served to generate the percentages presented above. See footnotes in Tables 1, 2 and 3 for the list of territories included in each region. *Source* : FAOSTAT (2020) and calculations for this study.

Total production of RTBs in developing countries averaged 841 million metric tonnes (t) in 2016–18 up from 244 million t in 1961–63 (FAOSTAT, 2020). Cassava and potato output each increased by roughly 200 million t; banana expanded nearly 100 million t and yam by 67 million t (FAOSTAT, 2020). Sweetpotato output rose and then fell for net decline of 5 million t; plantain production expanded by 25 million t (FAOSTAT, 2020). These distinct production patterns resulted in major changes in the respective shares of total output of RTBs in developing countries over time (Fig. 1) and perhaps more importantly at the regional level. Specifically, from 1988–90 to 2016–18, several RTBs had faster growth rates than many of the cereals in Africa, Asia and Latin America and the Caribbean (LAC) attesting to their growing importance in developing country food systems.

Growth in output of RTBs was particularly strong in Africa increasing from 59 million t in 1961–63 to 351 million t 2016–18 as area harvested expanded from 11 to 42 million ha (FAOSTAT, 2020). Two‐thirds of that increase consisted of cassava and yam with cassava alone accounting for 138 million t or nearly half (FAOSTAT, 2020). Perhaps even more noteworthy, 220 million t of that increase came since 1988‐90 as many countries experienced continued rapid population growth and local agri‐food systems came under additional stress due to droughts, humanitarian crises or spikes in commodity prices for food imports. In Asia, output of RTBs swelled from 135 million to over 395 million t over the same 60‐year period (FAOSTAT, 2020). Potato production surged by 160 million t during the last six decades and banana by 56 million t. Strong demand for more vegetables and fruit, reflecting consumers' desire to diversify their diets, drove these increases. Cassava expanded by 64 million t. The bulk (70%) of that increase was concentrated in South‐East Asia (SEA) where booming exports of processed cassava products such as starch catalysed strong productivity growth and greater production even as area harvested declined slightly (Newby & Le, 2017; FAOSTAT, 2020). Alternatively, sweetpotato fell by over 50% off its all‐time high due entirely to developments in China. In LAC, output nearly tripled for banana, plantain and potato as total regional production of RTBs climbed from 48 to 94 million t from 1961–63 to 2016–18 (FAOSTAT, 2020). Over the same period, cassava grew modestly, sweetpotato stagnated and yam expanded but from a very small base.

As part of these global trends, producers of RTBs such as cassava, potato and yam became increasingly market‐oriented with sales for cash taking on growing importance to complement their more traditional role as food security crops. Informal domestic and cross‐border commerce in RTBs is common in developing countries. Formal international trade in RTBs remains largely confined to exports of cassava processed products from South‐East Asia (SEA) and of bananas from LAC along with a very modest volume, but lucrative trade in potato, yam and derived products. In addition, RTBs have enabled different countries in various instances to address domestic food and feed requirements and thereby save foreign exchange otherwise spent on imports.

## Cassava

### Global developments

**Figure 2:**
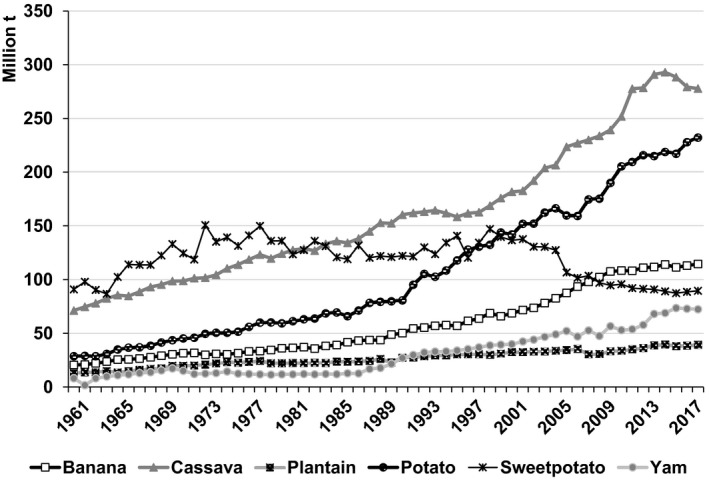
Annual production of banana, cassava, plantain, potato, sweetpotato and yam produced in developing countries, 1961–2018.^a a^FAOSTAT statistics for RTBs produced in developing countries in Africa, Asia and Latin America and the Caribbean (LAC) served to generate the data points presented above. See footnotes in Tables 1, 2 and 3 for the list of territories included in each region. *Source* : FAOSTAT (2020).

**Figure 3:**
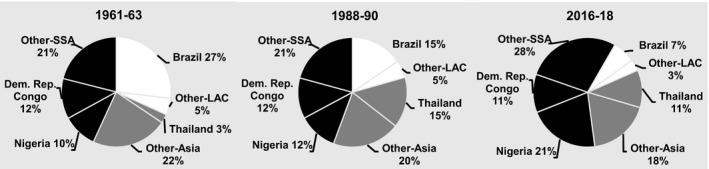
Percentages of total production of cassava in developing countries produced in different regions, subregions and countries in selected years, 1961–2018.^a a^FAOSTAT statistics for developing countries in Africa, Asia and Latin America and the Caribbean (LAC) served to generate the percentages presented above. See footnotes in Tables 1, 2 and 3 for the list of territories included in each region. SSA refers to sub‐Saharan Africa. *Source* : FAOSTAT (2020) and calculations for this study.

Between 1961–63 and 2016–18, cassava output increased from 75 to 282 million t – more than that of any RTB in the emerging economies (Fig. 2). Practically, all of that increase took place in sub‐Saharan Africa (SSA) with 138 million t and SEA (26%) with 58 million t in response to growing demand for both food and processed products (FAOSTAT, 2020). Output in LAC rose and then fell back to levels less than five million t higher than those in 1961–63 as growers switched to more remunerative crops. Hence, cassava production became increasingly concentrated in SSA (Fig. 3).

While total utilisation of cassava as food rose steadily in SSA, the evidence on use as animal feed is more mixed. FAOSTAT (2020) reports animal feed accounted for over 50% of total utilisation in some countries (e.g. Nigeria). Cassava is produced on small farms in SSA. Those same households often also raise small farm animals (e.g. goats, pigs, chickens) as a secondary activity using part of the cassava they harvest, usually peels and small roots, as animal feed (Oppong‐Apane, 2013). However, previous studies (Spencer & Ezedinma, 2017) suggest, field surveys (Odunze, 2019) found, and key informants recently indicated that percentage to be far lower than 50% of annual available supply. In Asia, output boomed in SEA driven by exports of dried cassava chips as feed initially, then starch and now a complex variety of distinct processed products (Cenpukdee *et al*., 1992; Parmar *et al*., 2017). In LAC, cassava utilisation historically has been more for human consumption and on‐farm use as animal feed than for processing into starch with the latter concentrated in Colombia, Brazil and Paraguay (Scott *et al*., 1992; Chuzel, 2001; Henry & Hershey, 2002; Demiate & Kotovicz, 2011; FAO, 2015). Recent trends have been more unsettled due to, among other contributing factors, the steady rise in the production and/or imports of substitutes such as maize and the decline in cassava output in Brazil, the region’s predominant producer.

### Africa

**Figure 4:**
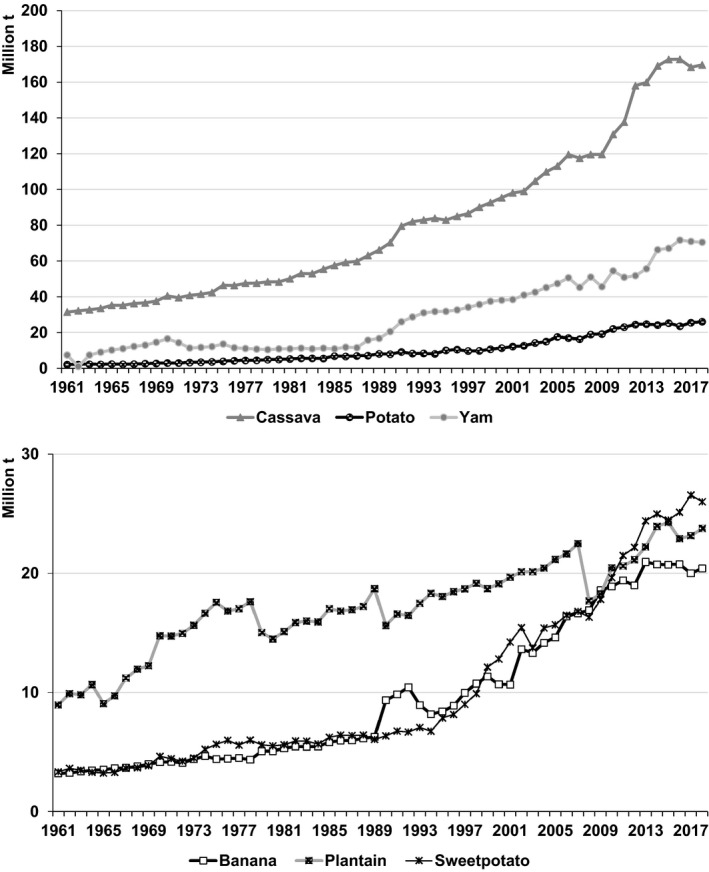
Annual production of banana, cassava, plantain, potato, sweetpotato and yam in Africa, 1961–2018.^a a^FAOSTAT statistics for production of RTBs produced in developing countries in Africa served to generate the data points presented above. See the footnote in Table 1 for a list of territories included in that region. *Source* : FAOSTAT (2020).

Brought to Africa from Brazil by Portuguese traders in the 1500s, cassava production surged in SSA over the last six decades going from 32 to 170 million t (Fig. 4). Area harvested expanded by 13 million ha (FAOSTAT, 2020). Over the last 30 years, total cassava output in SSA exceeded that of all the major food commodities while achieving more rapid growth rates (Table 1). Specifically, 67% of the increase in cassava output was concentrated in Nigeria, Democratic Republic of the Congo (DRC) and Ghana where it serves as the main staple or co‐staple. Angola, Mozambique, Malawi, Cameroon and Côte d'Ivoire accounted for another 16% of the increase since 1961–1963 (FAOSTAT, 2020). Several smaller of the 39 cassava‐producing countries in SSA also achieved noteworthy increases in output although smaller in relative terms, for example Burundi, Congo, Rwanda, Senegal, Tanzania and Zimbabwe (FAOSTAT, 2020).

**Table 1:** Average annual growth rates for food crops in Africa 1961–2018^a^

Crop	2016–18			Average annual growth rates (%)^b^								
	Production	Area	Yield	Production			Area			Yield		
	(000 t)	(000 ha)	(t ha^‐1^)	1	2	3	1	2	3	1	2	3
Cassava	170 275	18 670	9.1	2.7	3.4	3.1	1.5	2.9	2.2	1.2	0.5	0.9
Maize	79 122	39 495	2.0	3.1	2.6	2.8	1.7	1.7	1.7	1.4	0.9	1.1
Yams	71 038	8305	8.6	3.0	4.6	5.1	4.8	2.1	5.4	3.8	2.4	‐0.3
Rice, paddy	32 770	14 043	2.3	3.2	3.6	3.4	2.7	3.1	2.9	0.5	0.5	0.5
Sorghum	29 199	29 943	1.0	1.0	2.6	1.8	1.1	1.8	1.5	‐0.1	0.7	0.3
Wheat	26 445	10 064	2.6	2.7	2.5	2.6	0.3	0.7	0.5	2.4	1.8	2.1
Sweetpotato^c^	25 885	4557	5.7	2.2	5.2	3.7	2.7	4.4	3.6	‐0.5	0.7	0.1
Potato^c^	25 021	1828	13.7	4.9	4.3	4.6	3.7	3.4	3.6	1.1	0.8	1.0
Plantain	23 268	4139	5.6	2.2	1.1	1.6	1.8	0.8	1.3	0.4	0.3	0.3
Banana	20 386	2000	10.2	3.0	3.8	3.4	2.6	2.3	2.4	0.4	1.4	0.9
Beans, dry	6756	7599	0.9	3.0	3.9	3.5	2.3	3.2	2.7	0.7	0.7	0.7

Source: FAOSTAT (accessed in January–February 2020) and calculations for this study.

^a^ For this study, Africa includes North, South, East, Central and West Africa. Following FAO's classification, North Africa refers to Algeria*, Egypt*, Libya*, Morocco*, Tunisia* and Western Sahara*. South Africa consists of Botswana*, Eswatini*, Lesotho*, Namibia* and the Republic of South Africa*. East Africa is made up of Burundi, Comoros, Djibouti*, Eritrea*, Ethiopia*, Kenya, Madagascar, Malawi, Mauritius, Mozambique, Rwanda, Seychelles, Somalia, Sudan*, South Sudan, Uganda, United Republic of Tanzania, Zambia and Zimbabwe. Central Africa includes Angola, Cameroon, Central African Republic, Chad, Congo, Democratic Republic of the Congo, Equatorial Guinea, Gabon and ṇSao Tome and Principe. West Africa covers Benin, Burkina Faso, Cabo Verde*, Côte d'Ivoire, Gambia, Ghana, Guinea, Guinea‐Bissau, Liberia, Mali, Mauritania*, Niger, Nigeria, Senegal, Sierra Leone and Togo. * FAOSTAT reports these territories produced no cassava in 2016–2018.

^b^ 1 = 1988‐90 vs 1961–63; 2 = 2016–18 vs 1988–90; 3 = 2016–18 vs 1961–63, where the average annual growth rate is calculated as follows: $\left[{\left(\frac{\text{Ending}\;3‐\text{year}\;\text{average}}{\text{Beginning 3}‐\text{year average}}\right)}^{\frac{1}{\text{Number of years between beginning and end mid}‐\text{points}}}‐1\right]\times 100$

^c^ Data utilised to calculate the growth rates include revised annual estimates for Malawi based on Scott *et al*. (2013a, 2013b).

Expansion in cassava output was particularly noteworthy in Nigeria where over 80% of the 51.2 million t increase between 1961–63 and 2016–18 took place *after* 1988–90 (Nweke, 2005; FAOSTAT, 2020). Key demand‐side drivers of Nigeria's noteworthy increase in cassava output include (i) continued, very rapid (2.5% yr^‐1^ in 2019) population growth; (ii) the doubling of total population from 95 to over 200 million between 1990 and 2019 (UN, 2019); and, by implication, the run‐up in population density with growers seeking out those crops that yield the equivalent of more calories ha^‐1^ to meet growing household food requirements despite declining farm size (Spencer & Ezedinma, 2017). In addition, Nigeria's population went from 30% urbanised in 1990 to over 50% in 2018 (World Bank, 2019) generating growing urban demand for low‐cost, easy‐to‐prepare and consume food items. Similar developments, albeit on a smaller scale, took place across much, but not all, of SSA (Nweke, 2005; Spencer & Ezedinma, 2017).

On the supply side, cassava production in Nigeria resulted primarily from the 5.5 million ha expansion in area harvested between 1961–63 and 2016–18 as after 1988–90 growth in yields slowed considerably (Table 1). Cassava thrived in SSA as it adapts well to poor tropical soils where other crops struggle or fail; is easily propagated by stem cuttings in lieu of seed; resists drought, except at planting time; and is less susceptible to damage from locusts, thereby making it a good famine reserve crop as well (Nweke *et al*., 2002). In addition, widespread adoption of improved varieties and local processing technology made cassava that much more attractive as higher yields meant more food per area harvested some of which could be sold for cash (Nweke, 2005; Spencer & Ezedinma, 2017). Government support for dissemination of newly bred cassava cultivars and diffusion of small‐scale processing equipment to generate value‐added products was particularly catalytic in Nigeria and later in Ghana (Nweke *et al*., 2002; Odunze, 2019).

Cassava production also expanded as it plays multiple roles in SSA agri‐food systems including a rural food staple, source of cash income, famine reserve crop, urban food staple and with increased interest in its potential for animal feed in processed form, industrial uses and a source of foreign exchange (Spencer & Ezedinma, 2017). That cassava lends itself to diverse forms of food preparation also facilitated its spread across SSA and typically involves some form of processing of the root because of its rapid deterioration after harvesting. This also serves to eliminate the cyanogen present in higher concentrations in bitter than sweet cultivars (Spencer & Ezedinma, 2017).

Besides fresh and dried roots, cassava‐based foods include (i) pasty products made from soaking, fermenting and crushing the roots, with unsteamed wet cassava paste being particularly popular in West Africa; (ii) granulated products made by peeling, soaking, fermenting and sieving the roots, and then toasting the remaining pulp which is then often sold as *gari* most notably in much of West Africa; and (iii) cassava leaves prepared by leaching, and then pounding them into a pulp with a pestle and mortar before boiling in water with peanuts, meat or fish. The leaves are high in protein as well as provitamin A and vitamin C (Parmar *et al*., 2017) often deficient in SSA diets.^1^

The prospects of cassava for cash were further enhanced with the development and diffusion of improved, small‐scale processing equipment (Oppong‐Apane, 2013; Spencer & Ezedinma, 2017). Cross‐border trade in cassava and cassava products is common, particularly in West Africa. International commerce to the EU and beyond remains a future objective as strong domestic demand and high relative costs (e.g. average yields in SSA are less than half those in SEA, Table 2) tend to discourage exports.

**Table 2:** Average annual growth rates for food crops in Asia 1961–2018^a^

Crop	2016–18			Average annual growth rates (%)^b^								
	Production	Area	Yield	Production			Area			Yield		
	(000 t)	(000 ha)	(t ha^−1^)	1	2	3	1	2	3	1	2	3
Rice, paddy	683 499	143 070	4.8	3.2	1.5	2.3	0.7	0.4	0.5	2.4	1.1	1.8
Maize	361 208	67 980	5.3	5.0	4.0	4.5	1.3	2.0	1.7	3.6	2.0	2.8
Wheat	329 695	99 020	3.3	5.3	1.9	3.6	1.2	0.6	0.9	4.0	1.3	2.6
Potato	180 478	9195	19.6	4.1	4.1	4.1	2.6	2.5	2.6	1.5	1.5	1.5
Cassava	83 066	3844	21.6	3.9	1.6	2.7	2.1	−0.1	0.9	1.8	1.8	1.8
Banana	62 577	2301	27.2	3.8	4.3	4.1	2.0	1.9	1.9	1.8	2.4	2.1
Sweetpotato	59 691	3087	19.3	1.0	−2.2	−0.7	−1.6	−3.1	−2.4	2.6	0.9	1.7
Beans, dry	14 389	19 801	0.7	1.5	2.2	1.8	0.6	1.4	1.0	0.9	0.8	0.8
Sorghum	7743	7009	1.1	0.3	−3.0	−1.4	−1.5	−3.2	−2.4	1.9	0.3	1.0
Plantain	4977	407	12.2	0.9	3.4	2.2	−0.1	2.3	1.1	1.0	1.1	1.0
Yams	400	26	15.5	1.1	1.6	1.4	0.3	1.0	0.7	0.8	0.6	0.7

Source**:** FAOSTAT (2020) and calculations for this study.

^a^ For this study, Asia includes Central Asia, East Asia, South‐East Asia, South Asia, West Asia and Oceania. Following FAO's classification: Central Asia is made up of Kazakhstan, Kyrgyzstan, Tajikistan, Turkmenistan and Uzbekistan. East Asia consists of People's Republic of China, Democratic People's Republic of Korea, Mongolia and Republic of Korea. South‐East Asia is made of Brunei Darussalam*, Cambodia*, Indonesia, Lao People's Democratic Republic, Malaysia*, Myanmar, Philippines, Singapore*, Thailand, Timor‐Leste and Viet Nam. South Asia consists of Afghanistan, Bangladesh, Bhutan, India, Iran, Maldives*, Nepal, Pakistan and Sri Lanka. West Asia consists of Armenia, Azerbaijan, Bahrain, Cyprus, Georgia, Iraq, Jordan, Kuwait, Lebanon, Oman, Palestine, Qatar, Saudi Arabia, Syrian Arab Republic, Turkey, United Arab Emirates and Yemen. Oceania consists of Fiji, Kiribati*, Papua New Guinea*, Samoa*, Tonga, Tuvalu and Vanuatu*. * FAOSTAT reports these territories produced no potatoes in 2016–2018.

^b^ 1 = 1988–90 vs 1961–1963; 2 = 2016–18 vs 1988–90; 3 = 2016–18 vs 1961–63, where the average annual growth rate is calculated as follows: $\left[{\left(\frac{\text{Ending}\;3‐\text{year}\;\text{average}}{\text{Beginning 3}‐\text{year average}}\right)}^{\frac{1}{\text{Number of years between beginning and end mid}‐\text{points}}}‐1\right]\times 100$

By 2016–18, cassava production in SSA (Table 1) had already exceeded the baseline projection for output in 2020 of 164 million t as estimated by Scott *et al*. (2000). Moreover, over the last 30 years growth rates for area harvested exceeded those for maize, wheat and sorghum (Table 1). The surge in output that took place since 1988–90 was driven in large part by stronger demand from rising per capita incomes than previously anticipated (Scott *et al*., 2000), but confirmed in household consumption studies (Spencer & Ezedinma, 2017). Successful biological control of cassava mealy bug and the development and diffusion of improved varieties enabled a sustained supply response in terms of improved productivity even as area harvested expanded rapidly. Given current growth rates that expansion seems certain to surpass more optimistic, high demand projection of 184 million t in 2020 as well (Scott *et al*., 2000).

### Asia

Developments in the cassava sector in Asia were markedly different from those in SSA in several respects. Most cassava production in SEA serves as an industrial cash crop (Le *et al*., 2019). Over the last six decades, cassava production was highly concentrated in three countries – Thailand, Indonesia and Vietnam – in the SEA subregion. Together, they accounted for over 70% of total Asian output and 69% of the 64 million t increase in cassava production 1961–63 to 2016–18 (Howeler & Maung Aye, 2014; FAOSTAT, 2020).

Historically, cassava was processed in Thailand into dried chips for animal feed, and then shipped to the EU based on special trade quotas. As testimony to their competitiveness, Thai processors switched to pellets for easier handling and final use (Cenpukdee *et al*., 1992) only to revert to chips once the European market disappeared due to revisions to EU agricultural policy. Vietnam also became engaged in the business of exporting cassava. With EU policy changes and opportunities elsewhere, cassava processing pivoted into industrial products such as starch for export to China, other countries in East Asia and the United States for a variety of end uses (Parmar *et al*., 2017). In 2016, Vietnam alone exported US$ one billion of cassava and cassava products (Le *et al*., 2019). As the multi‐billion dollar yr^‐1^ market for starch continued to expand and diversify (Newby & Le, 2017), it induced growers and processors to participate by adopting improved production technology for cassava leading to sustained growth rates in productivity among the highest for all crops in Asia (Table 2). Since 2006, however, Indonesia witnessed a 40% decline in area due to weak government support and stiff competition in export markets due to lower yields, hence higher costs (Newby & Le, 2017; FAOSTAT, 2020).

Within SEA and other parts of Asia, cassava also serves as an important food crop (e.g. India, Vietnam) and in subnational locations where little output is sold (Newby & Le, 2017; Le *et al*., 2019). In several of those cases, trends were markedly mixed. India experienced a 50% decline in output since 2009 while production in Laos soared (FAOSTAT, 2020) but at more modest levels.

### Latin America and Caribbean

Brazil, once the world’s leading producer, traditionally dominates cassava production and use in LAC accounting for 65–75% of regional output (FAOSTAT, 2020). Paraguay accounted for another 5–6% for decades as well. Since 2006, both countries saw production contract by 30% or more with similar declines in Bolivia, Venezuela, Panama and Nicaragua as growers switched to more profitable crops (FAOSTAT, 2020). In Paraguay, 80% or more of production traditionally goes to on‐farm consumption and sales in the domestic market. The fall‐off in cassava output there suggests (a) local plants focused on foreign markets were left with excess capacity; (b) exports of starch slowly deteriorated; and (c) the resulting industry shake‐out forced firms to become more competitive to survive (Fretes, 2010). In that context, the recent uptick in cassava output is harder to assess.

Latin America and the Caribbean also saw a combined 2.2 million t expansion of cassava production in Colombia and Peru and spurts, albeit on a smaller scale, in Cuba and Haiti. However, the agro‐export boom, political instability and pockets of years of recurrent drought together with rapid increases in the production of substitutes such as maize (Table 3) all contributed to cassava’s decline in much of LAC in recent years. These countervailing tendencies resulted in a 4.7 million t (20%) increase in regional output and a 227 000 expansion in area since 1961–63 (FAOSTAT, 2020). Given more robust increases in Africa and Asia, the net effect was a decline in LAC’s share of total cassava production in developing countries (Fig. 3).

**Table 3:** Average annual growth rates for food crops in LAC 1961–2018^a^

Crop	2016–18			Average annual growth rates (%)^b^								
	Production	Area	Yield	Production			Area			Yield		
	(000 t)	(000 ha)	(t ha^−1^)	1	2	3	1	2	3	1	2	3
Maize	171 518	35 487	4.8	2.7	4.4	3.6	0.8	1.1	1.0	1.8	3.3	2.5
Banana	29 978	1199	25.0	2.1	1.7	1.9	1.7	0.5	1.1	0.4	1.1	0.8
Wheat	28 794	9123	3.2	2.6	1.1	1.8	1.1	−0.5	0.3	1.5	1.6	1.6
Cassava	28 478	2210	12.9	1.0	−0.3	0.3	1.1	−0.6	0.2	−0.1	0.3	0.1
Rice, paddy	27 598	4980	5.5	3.0	1.5	2.2	1.6	−1.4	0.1	1.4	2.8	2.1
Potato	20 161	1051	19.2	2.2	1.7	2.0	0.0	0.2	0.1	2.2	1.5	1.9
Sorghum	10 560	3359	3.1	5.9	0.1	2.9	3.7	−0.4	1.6	2.1	0.5	1.3
Plantain	10 416	986	10.6	2.4	1.7	2.0	2.2	0.7	1.5	0.2	1.0	0.6
Beans, dry	5839	6272	0.9	1.4	1.0	1.2	1.7	−1.0	0.3	−0.3	2.1	0.9
Sweetpotato	2895	281	10.3	−1.3	1.5	0.1	−0.9	0.2	−0.3	−0.5	1.3	0.4
Yams	1459	150	9.7	1.7	2.7	2.2	1.5	1.7	1.6	0.1	1.0	0.6

Source: FAOSTAT (2020) and calculations for this study.

^a^ For this study, LAC includes Mexico, the Caribbean, Central America and South America. Following FAO's classification: the Caribbean is made of Anguilla, Antigua and Barbuda, Bahamas, Barbados, Cuba, Dominica, Dominican Republic, Grenada, Haiti, Jamaica, Saint Kitts and Nevis, Saint Lucia, Saint Vincent and the Grenadines, Trinidad and Tobago; Central America consists of Belize, Costa Rica, El Salvador, Guatemala, Honduras, Nicaragua and Panama. South America consists of Argentina, Bolivia, Brazil, Chile, Colombia, Ecuador, Guyana, Paraguay, Peru, Suriname, Uruguay and Venezuela.

^b^ 1 = 1988–90 vs 1961–63; 2 = 2016–18 vs 1988–90; 3 = 2016–18 vs 1961–63, where the average annual growth rate is calculated as follows: $\left[{\left(\frac{\text{Ending}\;3‐\text{year}\;\text{average}}{\text{Beginning 3}‐\text{year average}}\right)}^{\frac{1}{\text{Number of years between beginning and end mid}‐\text{points}}}‐1\right]\times 100$

### Summary

During the last 60 years, SSA saw a 134 million t increase in cassava production resulting primarily from a 13 million ha expansion in area harvested (FAOSTAT, 2020). Over 75% of that increase in output occurred after 1988–90 (Fig. 4). Strong and growing demand for cassava and its derived products due to rapid population growth and urbanisation catalysed the adoption of improved technology to produce more food. They also led to more widespread pursuit of cash incomes from cassava sales at the farm level and by processors and traders off the farm. In SEA, output surged by 58 million t as exports of feed, then starch and now an array of distinct processed products for different end uses fomented the expansion, while tendencies in areas dominated by subsistence production were more mixed. In LAC, countervailing trends resulted in a 4.7 million t overall increase in production. As a result of these different trends, the location of cassava production experienced a major realignment with output now concentrated in SSA (Fig. 3). Moreover, by 2016–18 cassava output in developing countries had already exceeded baseline projections for 2020 with the more optimistic projection of 290 million t within easy reach (Scott *et al*., 2000).

## Potato

### Global developments

**Figure 5:**
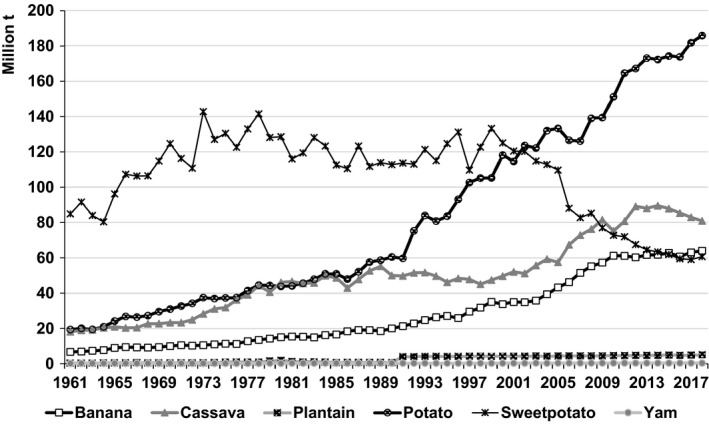
Annual production of cassava, potato, sweetpotato, banana, yam and plantain in developing countries in Asia, 1961–2018.^a a^FAOSTAT statistics for developing economies in Asia served to generate the data points presented above. See the footnote in Table 2 for a list of territories included in that region. *Source* : FAOSTAT (2020).

From 1961–63 to 2016–18, potato production in developing countries expanded by nearly 200 million t (FAOSTAT, 2020). In Asia alone, potato output increased from 19.7 to 180.5 million t (Fig. 5) – the biggest increase in production for any RTB in any region worldwide over that period. Elsewhere potato production in Africa expanded by 23 million t (Table 1; Fig. 4). Output in LAC rose 13 million t (FAOSTAT, 2020; Table 3), but with divergent trends within the region (Scott, 2011a). As a result, potato accounted for nearly 30% of the increase in RTB production for all developing countries since 1961–63 (FAOSTAT, 2020), thereby consolidating its position as the second leading RTB in developing countries (Fig. 1).^2^

### Asia

**Figure 6:**
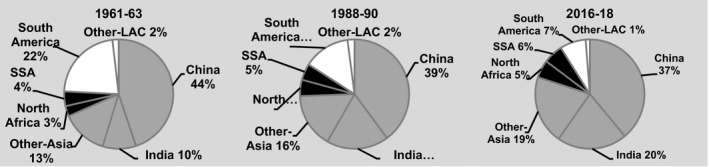
Percentages of total production of potato in developing countries produced in different regions, subregions and countries in selected years, 1961–2018.^a a^FAOSTAT statistics for production of potato in developing countries in Africa, Asia and Latin America and the Caribbean (LAC) served to generate the percentages presented above. See footnotes in Tables 1, 2 and 3 for the list of territories included in each region. SSA refers to sub‐Saharan Africa. *Source* : FAOSTAT (2020) and calculations for this study.

Driven by demand, potato became a more predominantly Asian crop over the last six decades (Fig. 6) as growth rates since 1988–90 were double or more those for rice, wheat and sorghum (Table 2). While of Peruvian origin, the expansion of potato production in China (75 million t), India (44 million t) and Bangladesh (9.4 million t) accounted for 80% of the increase in Asia.

As incomes increased across Asia over the last sixty years, consumers sought to diversify their diets away from rice (Pingali, 2015). In India and Bangladesh, additional factors contributing to the steady rise in per capita potato consumption included the tuber's neutral taste and gastronomic versatility; its nutritional attributes as a source of vitamins and minerals; the massive expansion of cold storage facilities enabling continuous in‐take throughout the year; and the strong vegetarian tradition in South Asian diets (Reardon *et al*., 2012; Scott *et al*., 2019b). Only very minor quantities serve as feed. Similar increases in consumption in China meant 80% of output went to bolster on‐farm consumption in the poorer, inland provinces and sales of fresh tubers for cash in urban markets (Scott & Suarez, 2012b). Although use of potato for feed in China declined in recent years to less than 20% of total utilisation, it rose in volume terms as production accelerated (FAOSTAT, 2020). Small, geographically isolated producers simply adjusted their potato utilisation practices to account for the booming demand for meat. Furthermore, unlike in many Western industrialised countries, the potato in Asia is typically not a cheap, starchy staple (Scott, 2002), nor as of yet is it consumed in any significant quantities in processed form, but rather as a complementary vegetable (Scott & Suarez, 2012a, 2012c).

Potato became increasingly attractive to many farmers in South Asia and China from an agronomic perspective as well. In India and Bangladesh, the crop flourished partly due to its very brief vegetative cycle of 90–110 days, shorter than the cereals. Additional agronomic attributes include its ability to produce more calories ha^‐1^ day^‐1^ and more calories unit^‐1^ of water than any other major food crop (Scott *et al*., 2019a).

Potato production also expanded rapidly in Asia because in the agri‐food system it contributes to food security for different actors in different ways. As the potato harvest in India and Bangladesh occurs in the lean period of food availability during the annual agricultural calendar, the tuber serves as an important supplementary source of food for growers and their households (Scott *et al*., 2019a). In the months following the main harvest in India, potato output acts as a backup to local food supplies for non‐producers in the form of stocks held in cold stores. Furthermore, given the high yields (Table 2), potato generates a timely source of income as well for the millions of small (<1 ha) farmers that dominate the potato sector in South Asia. In India, the potato also provides an annual source of nearly 300 million labour days for the massive number of rural workers – 75% of whom are women – employed in potato cultivation and harvesting (CPRI, 2015). Such employment enables farm workers in Bangladesh and India – many of whom are landless – to purchase and/or have access to food through their earnings or payments in kind, or both.

Exports and imports of seed, fresh potatoes and frozen French fries are a tiny share of potato output in Asia, but lucrative niche markets exist for tubers such as from Pakistan and Egypt to the Gulf States and China to SEA, for French fries to China and Singapore from industrialised countries and for seed to parts of Central Asia (Scott & Suarez, 2012a, 2012b; Anonymous, 2017).

### Africa

Cultivated by 40 countries across the region, strong growth in potato output involved two distinct systems. Egypt, Morocco, Rep. of South Africa and Algeria are all countries with irrigated production. Alternatively, in countries such as Nigeria and Tanzania in SSA, potato cultivation is rain‐fed. Together, these six nations accounted for over 65% of the regional increase in production from 2.1 to 25 million t since 1961–63 (FAOSTAT, 2020; Table 1). Similarly, in North Africa, potato serves a dual role as a food crop and an important source of foreign exchange from shipments to European markets in winter, while in the Rep. of South Africa domestic sales to urban markets and shipments to neighbouring countries predominate. By way of contrast, in SSA the crop serves as a complementary vegetable, household food security crop, source of cash and has mitigated the need for food imports in various instances, for example maize in Malawi (Scott *et al*., 2013a, 2013b). Potato’s high average yields – six times those of maize, and shorter duration (100 days) than other field crops as well make it particularly attractive (Table 1). With growing urbanisation in SSA, per capita potato consumption continues rising in many countries as potato trade expands in domestic markets (Scott *et al*., 2013a, 2013b). The increasing production and demand for potato in SSA have encouraged an increasing number of private seed companies, mainly Dutch, to establish seed programmes catering to the needs of African producers.

### Latin America and Caribbean

**Figure 7:**
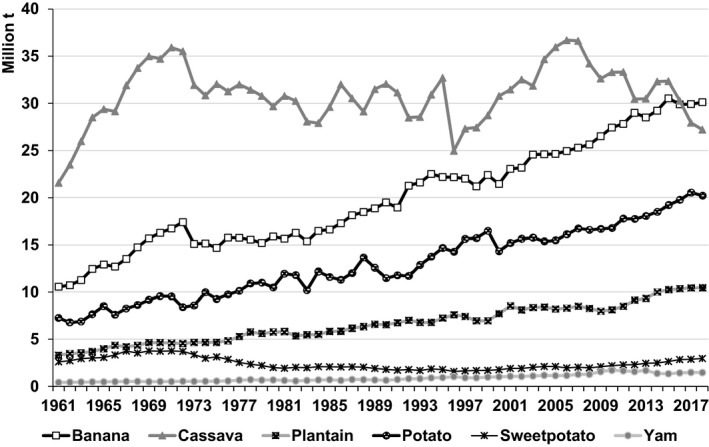
Annual production of banana, cassava, plantain, potato, sweetpotato and yam in LAC, 1961–2018.^a a^FAOSTAT statistics for production of RTBs produced in developing countries in LAC served to generate the data points presented above. See the footnote in Table 3 for a list of territories included in that region. *Source* : FAOSTAT (2020).

As the centre of origin for the crop, LAC witnessed not only a nearly threefold increase in from 7 to 20 million t from 1961–63 to 2016–18 (Fig. 7), but also a pattern that was highly uneven (FAOSTAT, 2020). With diffusion of improved cultivars, rising incomes and more eating out (Scott & Ocampo, 2013) combined with a re‐birth in consumer interest in Andean food commodities, Peru saw output quadruple over the last three decades. In contrast, Ecuador experienced negligible growth and since 2012 a 50% decline in area as interest in potato waned on and off the farm (Scott, 2011a). These trends also reflect the sharp differences in regional consumption patterns. Argentina and Chile saw relatively high per capita consumption (>60 kg yr^‐1^) decline significantly from much higher levels or stagnate as with rising incomes consumers diversified their diets, while growers switched to more profitable crops. In contrast, Brazil and Mexico with much lower initial per capita consumption (7–10 kg yr^‐1^) saw rising per capita incomes catalyse opposite trends (Scott, 2011b). Domestic and intraregional trade in potatoes and potato products continued to expand and the latter diversify as did imports including frozen French fries including from other countries in LAC where processing plants have been built (Scott & Ocampo, 2013). At the same time, increased domestic potato production reduced the need for food imports in times of crisis (Scott, 2011a).

### Summary

Since the late 1980s, Asia became the centre of global potato output. In Asia, potato had the highest rate of increase in area of any staple crop in the period since 1961 (Table 2); in Africa, it was second only to yam, albeit at a third the level of output (Table 1). Demand for potato remained particularly strong in much of South Asia and China, as rising incomes led consumers to diversify their diets away from rice. Foreign trade remains lucrative, but a negligible share of total output, for example < 2% in India (Scott, 2011a; Scott & Suarez, 2012b; Scott *et al*., 2019). Domestic markets for potatoes boomed particularly in Asia where tubers are produced increasingly for cash. Given these various developments – particularly in Asia, by 2016–2018 potato production in developing countries had already surpassed the most optimistic projections for 2020 by 27 million t as the bulk of the increase came from increases in area harvested and not yields as previously anticipated (Scott *et al*., 2000).

## Banana and plantain

### Global developments

Of SEA origin (Lescot, 2020), banana and plantain constitute perhaps the most complex food group among the RTBs. Production consists of (i) sweet or dessert bananas of two major varietal types Cavendish and Gros Michel with area harvested predominately of the former; (ii) cooking bananas broadly separated into East African highlands cooking and beer‐making bananas (EAHB); and (iii) plantain (Lescot, 2020). While plantain may also be rightfully considered as a type of cooking banana (Lescot, 2020), FAOSTAT (2020) lists plantain production and use as a separate commodity and hence is treated as such for this review. Nevertheless, for any given country, differences in opinion exist about which and how much of the three categories of banana previously mentioned may or may not be accurately reflected in FAO data for banana.

**Figure 8:**
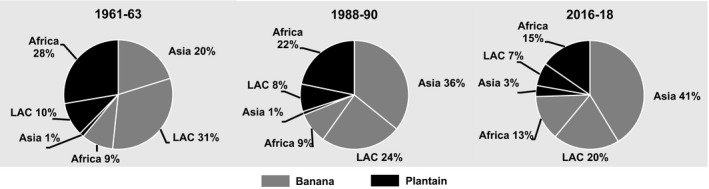
Percentages of total production of banana and plantain in developing countries produced in different regions, subregions and countries in selected years, 1961–2018.^a a^FAOSTAT statistics for production of banana and plantain produced in developing countries in Africa, Asia and Latin America and the Caribbean (LAC) served to generate the percentages presented above. See footnotes in Tables 1, 2 and 3 for the list of territories included in each region. SSA refers to sub‐Saharan Africa. *Source* : FAOSTAT (2020) and calculations for this study.

Banana production in developing countries rose to 122 million t during the last three decades (FAOSTAT, 2020), the largest share (56%) of output shifted from LAC to Asia (Fig. 8). Africa harvests less than 20% of the developing country total. In the process, banana became the third most important RTB in the world surpassing sweetpotato in terms of total production (Fig. 2).

Conversely, the largest share (60%) of global plantain production of 30 million t takes place in SSA (Table 1; Fig. 4). While plantain overtook sweet potato decades ago in LAC (Table 3; Fig. 7), it remains a very minor crop in Asia (Table 2). As bananas and plantain are perennials, they serve as the backbone of many farming systems producing the year‐round, protecting the soil from erosion and capable of surviving floods, drought and civil conflict. Often planted in association with several other crops, they also provide dietary diversity. Cooking bananas and plantain remain an important source of calories for many small farm households (Brown *et al*., 2017) particularly in SSA and parts of South Asia, SEA and LAC.

### Asia

India’s banana harvest over the last six decades reached 30 million t in 2016–2018 or 50% of the regional total for the 37 banana‐producing countries (FAOSTAT, 2020). Eighty‐five per cent of the 27 million t increase in India took place after 1988‐90 in response to rising incomes and growing demand for more fruit in the average diet. In so doing, India became the world’s largest banana producer (FAOSTAT, 2020).

According to Lescot (2020), Indian total banana and plantain output in 2018 was actually split between (i) dessert (89%), (ii) highland‐type (EAHB) cooking bananas (7%) and (iii) plantain (4%) with nearly all of all three going almost entirely for national consumption. Simultaneously, China showed the most accelerated increase in banana production in Asia as output rose from 42 000 t in 1961–63 to over 11 million t by 2016–18 (FAOSTAT, 2020). Almost all of these were dessert bananas. Even so, China still imported nearly two million additional t of dessert banana (FAOSTAT, 2020) while setting up plantations in Cambodia, Laos and Myanmar to help satisfy rapidly growing internal demand (Grimsditch, 2017).

Indonesia (7.2 million t) and the Philippines (9.6 million t) are the other major banana producers in SEA with the latter exporting 45% of its production. According to Lescot (2020), roughly 30% of their banana output consists of cooking banana.

In contrast, plantain production in Asia remained much more modest both in terms of the number of countries (16) and total output of 1.5 million t. India (1.25 million t) alone harvested 83% of the regional total with over 84% for internal consumption (FAOSTAT, 2020).

### Africa

Banana witnessed increases in production (17.1 million t) and area (1.5 million ha) respectively in SSA between 1961–63 and 2016–18 (FAOSTAT, 2020; Table 1). Over half these increases were accounted for by Angola, Burundi, Kenya, Rwanda and Tanzania. Various types of banana are unique to Africa, and these can be eaten fresh, cooked, fried and processed to be served as baby food, juice and beer. People living in the highlands of central Africa (e.g. Rwanda) reportedly eat more bananas than anyone else in the world, deriving 35% of their daily calories from the crop (Stellenborsch, 2020).

EAHB are predominately cultivated in East and Central Africa; only 8% of West Africa’s banana production consists of cooking banana (Lescot, 2020). Cooking bananas are typically eaten boiled, fried or mashed. Case studies also found that in particular production pockets, the EAHB are often produced by small farmers and processed into beer, a popular, low‐cost alternative to traditional, grain‐based beer (MAL, 2012), thereby providing an extremely important – if not only – source of cash income for these poor households.

According to Kwa & Temple (2019), nearly all of SSA’s plantain production is harvested in West Africa and for on‐farm or domestic consumption; only 15% is for export. While plantain production in SSA increased by 13.7 million t and area by 2.1 million ha between 1961–63 and 2016–18 (FAOSTAT, 2020), growth rates including those for yields were among the lowest of all the major food crops in the region (Table 1).

### Latin America and the Caribbean

Once the leading region in global banana production, LAC’s output expanded at a far slower pace than in Africa or Asia for decades due to disease outbreaks, adverse weather, restrictions on exports and the very narrow genetic base of the dessert banana that dominates regional production (Southgate & Roberts, 2016; Lescot, 2020). Notwithstanding, leading producers in LAC such as Ecuador have a long history of producing dessert bananas that unlike Africa or Asia are overwhelmingly for export (Southgate & Roberts, 2016). Of the 30 banana‐producing countries in LAC, just five, Costa Rica (2.5), Guatemala (4.0), Brazil (6.7), Colombia (3.7) and Ecuador (6.4 million t), accounted for 78% of the region’s 29 million t of production in 2016–2018 (FAOSTAT, 2020). These same countries harvested 74% of the region’s 1.1 million ha in banana (FAOSTAT, 2020).

Cooking banana constitutes 24% (0.7 million t) of the Caribbean’s banana production of 3.0 million t, but less than 4% for the region as a whole (Lescot, 2020).

Plantain production in LAC averaged 10 million t in 2016–18 (FAOSTAT, 2020) and dominated by Colombia (2.0) and Peru (1.2 million t). Some 90% of all plantain harvested in LAC is for internal consumption.

### Summary

With strong domestic demand and vast internal markets, India and China emerged as major producers driving the ascendancy of banana among RTBs in developing countries and leading Asia to replace LAC as the centre of global production. Cooking banana and plantain dominate banana production in SSA where they serve for on‐farm consumption and sales, while 75% of LAC’s banana harvest consists of dessert banana almost mostly entirely for export and plantain largely for domestic consumption.

## Sweetpotato

### Global developments

**Figure 9:**
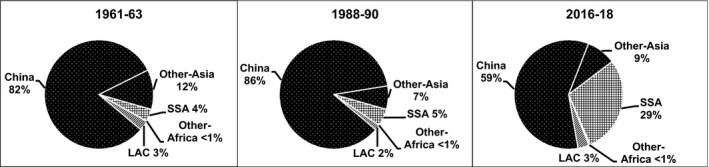
Percentages of total production of sweetpotato in developing countries produced in different regions, subregions and countries in selected years, 1961–2018.^a a^FAOSTAT statistics for production of sweetpotato produced in developing countries in Africa, Asia and Latin America and the Caribbean (LAC) served to generate the percentages presented above. See footnotes in Tables 1, 2 and 3 for the list of territories included in each region. SSA refers to sub‐Saharan Africa. *Source* : FAOSTAT (2020) and calculations for this study.

Though its origins lie in LAC, Asia and specifically China have long been the largest sweetpotato‐producing region and country in the world‐dominating global output like no other nation for any other RTB. However, over the last thirty years, China's share of developing country production fell (Fig. 9) as output there receded from 60‐year highs to half that total (Fig. 2). Over the same time, production in SSA took off expanding by 3.9 million ha and capturing nearly a third of developing country production (Fig. 9). Meanwhile, sweetpotato output in LAC stagnated as some countries saw noteworthy declines, while others witnessed production reboots (FAOSTAT, 2020). Consequently, sweetpotato shrank as a share of total developing country RTB production (Fig. 1) and fell behind banana in terms of total production (Fig. 2).

Sweetpotato roots, vines and leaf have long been utilised for multiple purposes in developing countries including flour, fodder, starch and different types of food products (Woolfe, 1992). Farm‐level use of sweetpotato in some form as animal feed has been particularly widespread (Scott, 1992). Breeding efforts in SSA and the release of improved varieties have renewed interest in further work in the development of processed sweetpotato products for that region (Andrade *et al*., 2009).

### Asia

Sweetpotato production and use in China evolved over roughly four distinct periods since 1961–1963. The *famine recovery* period 1961–1973 began marked by hunger and food shortages in the wake of the famines of 1959 and 1961 (Li *et al*., 1992; Gitomer, 1996). These dire conditions spurred a near doubling of sweetpotato output from 78.7 million t in 1961–1963 to 134 million t in 1973 even as area harvested fell from 10.9 to 9.5 million ha (FAOSTAT, 2020). Instead, the diffusion of improved technology drove up yields and production (Li *et al*., 1992; Table 2). The use of non‐edible parts for planting, low soil nutrient requirements, short cropping season and relatively low production risks made sweetpotato well‐suited for such situations (Mukhopadhyay *et al*., 2011; Reynolds *et al*., 2015).

During the ensuing *self‐reliance period* 1974 to 1988–90, the central government's policy focused heavily on boosting production of basic food grains such as rice and wheat (Alexandratos & Bruinsma, 2012). This also meant reducing area devoted to sweetpotato as it was not considered an 'essential crop' by policymakers (Li *et al*., 1992). As a result, sweetpotato production fell to 104.7 million t as area harvested imploded from 9.2 to 6.3 million ha (FAOASTAT, 2020). As grain production recovered, sweetpotato for direct human consumption became less attractive and instead its use as animal feed jumped from 14% of output in 1961–1963 to 42% in 1988–1990 (FAOSTAT, 2020).

In the *market adjustment* period 1990 to 1999, policy reforms gave growers greater freedom to respond to market signals allowing farmers to take advantage of shifting food preferences, for example the demand for more pork. Initially, these reform measures proved a boon to some sweetpotato producers. Village‐level hog production often involved the use of sweetpotato for pig feed (Li *et al*., 1992; Wiersema, 1992) as demand for pork, the Chinese preferred meat, grew much more rapidly than for sweetpotato for direct human consumption. This practice was particularly true in Sichuan province, in south‐west China, long the largest sweetpotato‐ and pork‐producing province and isolated from the then centre of maize production in the far northeastern part of the country. The combined effect saw annual sweetpotato production rebound to 126.1 million t (FAOSTAT, 2020).

With the *post‐globalisation* period from 1999 to present, China's economy became much more competitively oriented. In the process, livestock producers became bigger and more inclined to substitute industrialised feed sources based on readily transportable grains for more traditional inputs such as sweetpotato roots mixed with other farm and household remnants (Rae, 2008). Likewise, urban consumers' interest in new, easier to prepare, processed foods as well as a wider array of fresh vegetables dampened demand for fresh sweetpotato for direct human consumption. Economic development also led to the expansion of irrigation and rural road networks. Consequently, producers seized opportunities to produce and market other, more remunerative crops to serve newly emerging markets – as well as to adopt the technology increasingly available to do so. These same factors also contributed to sweetpotato output falling nearly by half to 59.7 million t between 1999 and 2016–2018 as area harvested imploded from 5.9 to 3.4 million ha (FAOSTAT, 2020). By 2015–17, more sweetpotato went for feed (49%) as those small‐scale growers who continued to cultivate the crop had fewer market outlets for human consumption and fewer resources to opt for purchase of alternative feeds.

Trends for sweetpotato production and use in much of South and SEA followed a similar pattern as in China. Output rose in the 1980s and early 1990s only to fall back to levels just 10% higher or still lower than those achieved in the 1960s for similar reasons mentioned above with certain possible exceptions. For example, India recorded a 40% increase in sweetpotato output since 2010 suggesting renewed interest but to levels achieved decades earlier (FAOSTAT, 2020) making that recovery harder to interpret.

### Africa

Sweetpotato evolved quite differently in SSA. Area harvested increased by 3.9 million ha and output by 22.4 million t. Average growth rates for production 1988–90 to 2016–18 were higher (5.4% yr^‐1^) than any other of the 11 major food commodities in the region (Table 1) and area harvested growth (4.5% yr^‐1^) second only to yams. These trends were all the more remarkable given the decades of neglect of sweetpotato's potential by national and international agricultural research organisations. Furthermore, while six countries, Malawi, Nigeria, Ethiopia, Angola, Uganda and Tanzania, accounted for 53% of the increase in output since 1961–1963, the expansion was remarkably widespread across the continent (FAOSTAT, 2020). An array of factors combined to induce the increase in area harvested and the prospect of sustained increases in the years ahead:

- rapid population growth (e.g. Nigeria, Rwanda);
- capability of sweetpotato to produce lots of food very quickly, cheaply and easily in times of severe shortages of basic staples, for example due to drought (Malawi) or humanitarian disaster (Rwanda) (Tanganik *et al*., 1999; Kapinga *et al*., 2005);
- crop's ability to produce more calories per hectare on poor, often degraded soils than cassava (Uganda, Tanzania) or yam (Nigeria) and in a shorter growing season with much lower labour and planting material requirements (Low *et al*., 2009);
- sweetpotato’s natural climate resilience, particularly against heat stress which has been further enhanced by genetic improvement programmes; and
- renewed interest in improved nutrition as a national development goal in Africa (Covic & Hendriks, 2016), supported the development, then diffusion of high‐yielding varieties with abundant beta‐carotene – a precursor of vitamin A (Woolfe, 1992) that led to several well‐documented instances of a significant reduction in chronic vitamin A deficiency (Low & Thiele, 2020).

### Latin America and the Caribbean

While growth rates for sweetpotato production and area harvested in LAC were essentially flat from 1961–63 to 2016–18 (Table 3), the aggregate statistics mask a distinctly dichotomous trend over space and time. Some countries such as Argentina and Brazil saw a major shift out of sweetpotato into more lucrative crops. Others including Cuba, Haiti and parts of Peru recorded a surge in production to meet local staple food requirements as agri‐food systems came under increasing stress due to natural disaster, deforestation and population pressure.

### Summary

Global trends for sweetpotato since 1961–1963 have been dominated by the rise and then sharp decline in production and use in China contrasting with the increase in output in SSA. While area harvested fell in China by 7.5 million ha, it expanded by 3.9 million ha in SSA (FAOSTAT, 2020). Similarly, while sweetpotato output in developing countries was only 57% of the baseline projection for 2020 (Scott *et al*., 2000), this result driven by trends in China masks the growing importance of the crop in SSA. Thus, as many agri‐food systems come under increasing pressure in developing countries due to climate change and population pressure in the decades ahead, sweetpotato may capture renewed interest as has occurred recently in SSA.

## Yam

### Global developments

**Figure 10:**
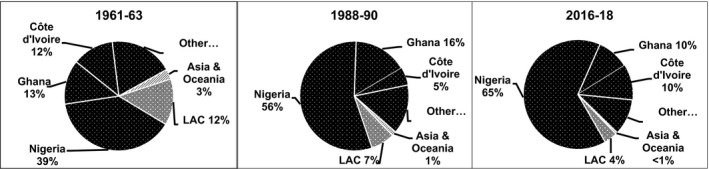
Percentages of total production of yam in developing countries produced in different regions, subregions and countries in selected years, 1961–2018.^a a^FAOSTAT statistics for yam produced in developing countries in Africa, Asia and Latin America and the Caribbean (LAC) served to generate the percentages presented above. See footnotes in Tables 1, 2 and 3 for the list of territories included in each region. SSA refers to sub‐Saharan Africa. *Source* : FAOSTAT (2020) and calculations for this study.

Yam production increased from 5.9 to 72. 9 million t between 1961–63 and 2016–18 – one of the largest increases in production of any RTB worldwide over the last 60 years (Fig. 2). Although yam includes roughly 600 species with some indigenous to West Africa, others to the Caribbean and still others to Asia (Asiedu & Sartie, 2010), 88% of that increase was concentrated in Côte d’Ivoire, Ghana and Nigeria (Fig. 10). According to FAOSTAT (2020), production and production increases were far more modest in LAC (1.4 million t in 2016–2018 and a production increase of one million t since 1961–63) and Asia including Oceania (383 000 t and an increase of 190 000 t).

### Africa

Of the 24 SSA countries that harvest yam, 90% of the overall production in SSA since 1961–1963 was harvested in Nigeria, Ghana and Côte d'Ivoire with Nigeria alone accounting for 68% of the total (Fig. 10). Benin and Togo also witnessed noteworthy increases in yam production as they too form part of the *yam belt* that stretches across the coast of West Africa from Côte d'Ivoire to Cameroon. Yam also expanded in East and Central Africa, but production there still accounts for less than 10% of the SSA total (FAOSTAT, 2020).

In West Africa, yam serves as a rural staple and source of cash income, an urban secondary food, a source of foreign exchange and an important component in traditional cultural/ceremonial activities that sets it apart from other commodities (Nweke, 2016; Frossard *et al*., 2017). Elsewhere in SSA, yam is more of a food security crop and is reportedly used in traditional medicine (Asiedu & Sartie, 2010).

A concerted research effort to improve yam productivity and use has lagged behind those of other RTBs in SSA (Asiedu & Sartie, 2010). Hence, over 80% of the aforementioned increase in output took place after 1988–90 (FAOSTAT, 2020). In the process, yam cultivation became much more noteworthy in SSA – particularly in Nigeria, Ghana and Côte d'Ivoire – trailing only maize and cassava in aggregate importance (Nweke, 2016; Table 1). Furthermore, the high demand for yam in urban markets – an estimated 60% of production is sold for cash, and for cross‐border trade and exports to Europe (CBI, 2019) and North America, continues to act as additional key drivers behind the surge in output in recent decades besides food for on‐farm consumption.

### Latin America and the Caribbean

Of the 47 yam‐ producing developing countries, 16 are in the Caribbean and 13 of those produce less than 10 000 t yr^‐1^. Among the principal producers in LAC, Haiti saw yam output nearly double to 439 000 t since 2006 to bolster urban food supplies with minor exports to elsewhere in the Caribbean and the United States (FEWS NET, 2018). Colombia also saw steady rise in yam output to over 400 000 t, while since 2012 Cuba experienced a 300 000 t collapse to 56 000 t in 2017 as production in Brazil remained flat (FAOSTAT, 2020). These countervailing tendencies ended up with yam output in LAC nearly doubling since 1961–63 while still equalling just 2% of total production for developing countries (Table 3; Fig. 10).

### Summary

Yam production surged in SSA over the last six decades driving the total for developing countries in 2016–18 (Table 4) beyond the 66.7 million t projected for 2020 (Scott *et al*., 2000). Eighty per cent of that increase came after 1988–1990 and in three West African countries (FAOSTAT, 2020). Those nations like those elsewhere in SSA have growing and increasingly urban populations, creating growing demand for increases in yam production.

**Table 4:** Production and area harvested of RTBs and annual average value of production in developing countries, 2016–18

n*	Crop	Production			Area					
		(000 000 t)			(000 000 ha)			Price US t^‐1^ ^a^	Value US$ billions	% of total
		1961–63	1988–90	2016–18	1961–63	1988–90	2016–18			
86	Cassava	74.6	150	281.8	9.9	15.1	24.7	128	36	10.7
90	Potato	28.8	79.1	225.7	3.6	6.3	12.1	318	72	21.2
96	Sweet potato	93.1	121	88.5	12.6	9.1	7.9	741	66	19.4
101	Banana	21.1	54.4	112.9	2	4.1	5.5	621	70	20.7
63	Yam	5.9	18.7	72.9	1.2	2	8.5	743	54	16.0
46	Plantain	13.4	24.5	38.7	2.5	4.2	5.5	597	23	6.8
88	Other RTBs^b^	6.8	9.5	21	1.3	1.7	3.1	920	18	5.3
	Total	243.6	457.4	841.0	33.1	41.8	67.3		339	100

n* denotes number of developing countries that produce that RTB (FAOSTAT, 2020).

^a^ IFPRI’s IMPACT model for commodity projections utilizes average prices (US$ t^‐1^) in 2005 of 515.99 for banana and plantain, 673.1 for Sweetpotato and yam, taro and yautia and 116.89 and 257.85 for cassava and potato, respectively. Estimated prices for 2030 are 735.61 for banana, 684.82 for plantain, 819.28 for yam, taro and yautia, 815.26 for sweetpotato, 384.17 for potato and 140.25 for cassava. Prices listed above are estimates for 2017 based on a linear interpolation of prices for 2005 and 2030, then rounded to the nearest dollar. The prices for 2005 and 2030 are based on IFPRI researchers’ determinations of past and future market tendencies as well as consultations with specialized scientists in international agricultural research centers around the world.

^b^ Other RTBs includes the production (in million t) of taro (10.2), yautia (0.5) and R&T, nes (10.3) for 2016–2018 and with a production value (US$ billions) of 7.6 for taro, 0.3 for yautia and 9.9 for R&T, nes in 2016–2018. Totals may not sum due to rounding. Source: Production and area (FAOSTAT, 2020); prices (IFPRI, 2019).

## RTBs in the aggregate

### RTBs in the past

Production of RTBs in developing countries increased by nearly 600 million t (nearly 250%) since 1961–63; 64% of that increase occurred after 1990 (Table 4). SSA accounted for 291 million t of that increase with 138 million t (47%) consisting of cassava and 61 million t (21%) of yam. Asia accounted for another 262 million t with 160 million t (61%) consisting of potato, 64 million t (24%) consisting of cassava and 56 million t (21%) banana, thereby offsetting the 27 million t decline in sweetpotato (FAOSTAT, 2020). For RTBs in Africa, Asia and LAC together, nearly half the 597 million t increase (43%) came from just three countries: Nigeria, China and India.

Area harvested in RTBs expanded by 34 million ha since 1961–1963 (Table 4). The vast majority (thirty million ha) took place in Africa: nearly 24 million ha was made up of cassava (13 million ha), yam (7 million ha) and sweetpotato (3.8 million ha). Nigeria alone accounted for 13.7 million ha (40%) of the overall increase in developing countries. Conversely, Asia recorded only a net 1.8 million ha expansion in area since 1961‐63. Increases in area harvested for potato (6.9 million ha), banana (1.5 million ha) and cassava (1.5 million ha) were largely offset by the 8.5 million ha decline in sweetpotato (FAOSTAT, 2020). These data underline the ongoing shifts in the location and importance of RTBs worldwide as growers continuously adjust to the changing opportunities and constraints that they are faced with shifting demand patterns, changes in government policy and the challenges consisting of evolving growing conditions such as climate change.

Utilisation patterns for RTBs have become increasingly more crop‐specific and even country‐specific. For cassava, 50% or more of annual output goes for feed use in Brazil joining Thailand, and Vietnam as countries where non‐food uses account for most of output. A similar trend emerged for sweetpotato in China. In contrast, potato, banana, yam and plantain are overwhelmingly utilised for human consumption.

Overall, in Africa since 1988–90, cassava and yam at much higher levels of output had faster growth rates in production than the cereals (Table 1). Banana, potato and sweetpotato albeit at much lower levels of output did the same (Table 1). In Asia, potato, banana and cassava also grew faster than rice, wheat or sorghum (Table 2). Similarly, in LAC growth rates for production since 1988–90 for potato, banana and plantain were higher than those for wheat, rice and sorghum (Table 3).

### RTBs at present

Based on estimated farm‐gate prices and FAO production data for 2016–2018, potato, cassava, sweetpotato, yam, bananas and plantain had an average annualized economic value of US$ 339 billion (Table 4). Potato (21.9%), banana (20.7%) and sweetpotato (19.4%) accounted for 62% of the total. With the lowest price t^‐1^, cassava’s share of total value is cut to less than a third of its share of total production. Furthermore, potato expanded faster than banana previously considered the most likely of the top four to grow fastest in the years ahead (Petsakos *et al*., 2019). Alternatively, given the far greater magnitude of cassava production in SSA (Table 1; Fig. 4) – the region widely recognised as facing the greatest challenges to meet future food requirements particularly of low‐income households, some observers have suggested other factors, for example the crop’s capacity to adopt to climate change (Jarvis *et al*., 2012) in addition to the estimated value of production be considered when ranking the importance of production of different RTBs.

### RTBs in the future

The most recently published estimates of global food production to 2030 and 2050 are those published by the International Food Policy Research Institute (IFPRI, 2019), a research centre long dedicated to these sorts of projections. Utilising the IMPACT model (Robinson *et al*., 2015), IFPRI (2019) projects roots and tubers (R&Ts) – the projections include bananas and plantain with fruits and vegetables – would reach 857 million t in 2030 and 995 million t in 2050 (Table 5).^3^

**Table 5:** Projections for R&T production, consumption and trade in developing countries in 2030 and 2050^a^

Region/commodity^b^	Production (million t)	Value (%)	Production (million t)		Consumption (kg capita^−1^ yr^−1)^			Net trade (million t)		
	2016–18		2030	2050	2010	2030	2050	2010	2030	2050
**World**			962	1101	65	67.7	68.9	0	0	0
Developing	**684**	100	857	995	65.8	69.5	71.1	5.6	−0.6	−0.6
Developed			105	106	61.2	57.5	56.1	−5.6	0.6	0.6
**Asia and Pacific** ^c^	**304**	**44**	356	379	46.9	48.3	45.6	−4.9	1.4	28.7
Potato	157	20								
Cassava	83	4								
Sweet potato	59	18								
Other R&Ts^d^	5	2								
**Ex‐Soviet Union** ^e^	**11**	2	10	12	n.a.	n.a.	n.a.	n.a.	n.a.	n.a.
Potato	11	2								
**Africa and M East** ^f^	**317**	**47**	361	485	109.3	113.8	116.9	−1.8	−16.5	−39.4
SSA	**294**	**45**	332	449	146.4	149	149.1	−1.1	−17.7	−42.8
Cassava	170	9								
Yam	71	21								
Potato	14	3								
Sweet potato	26	8								
Other R&Ts^d^	13	3								
N Africa & M East^f^	**23**	**4**	29	36	39	37	36.3	−0.8	1.2	3.4
North Africa	**11**	2								
Potato	11	2								
Middle East^f^	**12**	2								
Potato	12	2								
**LAC**	**52**	**7**	83	98	51.1	48.3	45.7	0.2	16.1	29.5
Cassava	28	2								
Potato	20	3								
Sweet potato	3	1								
Other R&Ts^d^	1	1								

^a^ These data are for R&Ts (Roots and Tubers) only; they do not include banana and plantain as the latter are included in Fruits and Vegetables in the IMPACT model. Roots and tubers include cassava, potato, sweetpotato, yams, and aggregated other roots and tubers. The data in bold are the sums for R&Ts for all developing countries or the equivalent for particular regions according to groupings used by IFPRI. Total production is aggregated across irrigated and rain‐fed systems at the national level and aligned with years as reported in FAOSTAT. Per capita food consumption is based on food availability at the national level. Net trade includes negative and positive numbers indicating that a region is a net importer or exporter, respectively, and balances to zero at the global level. Values reported for 2010 are calibrated model results. Projections for 2030 and 2050 assume changes in population and income as reflected in the IPCC's Shared Socioeconomic Pathway 2. Climate change impacts are simulated using the IPCC's Representative Concentration Pathway 8.5 and the HadGEM general circulation model. Further documentation is available at www.ifpri.org/program/impact‐model.

^b^ For specific commodities, only production over one million t are listed. As all commodity figures are rounded, regional and sub‐regional totals and sub‐totals may not sum.

^c^ Includes East, South. and Southeast Asia and Oceania as per FAOSTAT.

^d^ Other R&Ts includes taro, yautia, arracacha, mashua, olluco, yacon and other roots and tubers.

^e^ The ex‐Soviet Union consists of Central Asia and is made up of Armenia, Azerbaijan, Georgia, Kazakhstan, Kyrgyzstan, Tajikistan, Turkmenistan, and Uzbekistan.

^f^ North Africa includes Algeria, Egypt, Libya, Morocco, Tunisia and Western Sahara. Middle East consists of Bahrain, Cyprus, Gaza Strip, Iran, Iraq, Jordan, Kuwait, Lebanon, Oman, Qatar, Saudi Arabia, Syria, Turkey and United Arab Emirates. Source: 2016–2018 (FAOSTAT, 2020); 2030 and 2050 (IFPRI, 2019).

More specifically, the projection for R&Ts in 2030 for Asia including Oceania is 356 million t versus 304 million t in 2016–18 (including only East, South, South‐East Asia and Oceania to comply with IFPRI’s separation of Asia and the Pacific from their West Asia/North Africa region). The projection for SSA is 332 million t versus 294 million t in 2016–2018 (Africa data not including North Africa) that for LAC 82 million t versus 53 million t in 2016–18.

These production figures indicate that in 2016–18 R&T output is divided overwhelmingly between Asia and SSA with LAC accounting for only a minor (<10%) share. Based on economic value, SSA accounts for about half of the developing country total with yam, cassava and sweetpotato making up the predominate shares. Furthermore, given 2016–18 production levels and an annual growth rate for R&T production of under 1.5% over the next 13 years, the output of R&Ts in 2030 will surpass the IFPRI projections for R&Ts in Asia and SSA, less so for LAC. Hence, these IFPRI production estimates for 2030 appear too conservative.^4^

IFPRI (2019) projections translate into only minor changes in estimated future total per capita consumption of R&Ts (Table 5). In effect, population growth will offset the increase in output. Projections for future foreign trade to 2030 are also in line with historical tendencies. They indicate that with certain noteworthy exceptions, domestic demand will absorb the increases in production as also reported in recent crop‐specific studies (Scott & Kleinwechter, 2017; Scott *et al*., 2019).

### RTBs going forward

Following the agri‐food system framework, this section presents a concise review of those issues and opportunities that merit particular attention based on the literature review and data analysis carried out for this study. This overview is by no means intended to be exhaustive, nor exclusive, but rather one benchmark in the ongoing debate about achieving the full potential of RTBs for the benefit of the growing numbers of low‐income producers and consumers in developing countries in the future.

### Cassava

Various studies have pointed to the greater adaptability of cassava to climate change than other food crops currently cultivated in different developing country subregions (Jarvis *et al*., 2012; Rosenthal & Ort, 2012; El‐Sharkawy, 2014; Parmar *et al*., 2017). To capitalise on cassava’s capability to achieve greater productivity, more efficient production and distribution systems for improved planting material and better soil fertility management practices are frequently mentioned (Alene *et al*., 2018). In recent years, the tension between strong demand for food, feed and industrial products in combination with the advent of climate change and the expansion of area harvested into heretofore more marginal growing areas (Reynolds *et al*., 2015) have put added pressure on the sector to develop and diffuse high‐yielding varieties with high dry matter content and starch, with longer shelf life that are drought‐tolerant and pest and disease (cassava mosaic virus and in East Africa brown streak virus)‐resistant (Howeler & Maung Ha, 2014). Moreover, precisely because numerous processed products can be made from cassava: starch, flours, feed, plastics and bio‐fuels (Sanginga & Mbabu, 2015; Parmar *et al*., 2017); a better and more timely understanding of particular market developments will be increasingly important in assisting growers and processors in making production and marketing decisions and reducing risk (Newby & Le, 2017). Such information will also be key in making decisions about which processing equipment improvements need to be prioritised (Fretes, 2010) and along with market assessments in enhancing the probability of success of such initiatives.

### Potato

India and China’s massive populations and growing urbanisation – an estimated 40% of India’s population or nearly 600 million people will be living in urban areas by 2030 – portend increasing demand for food away from home including restaurants as well as in the form of snacks and convenience foods (CPRI, 2015) that together suggest strong prospects for continued growth in utilisation and output. Increased attention to more environmentally sustainable use of nitrogen fertiliser and pesticides, better soil management (e.g. by 'reseeding' fields with green manure), improved water‐use practices and in South Asia up‐grading cold storage infrastructure are key to achieving that outcome (Scott *et al*., 2019b; Gatto *et al*., 2020). In South Asia in particular, Petsakos *et al*. (2019) pessimism about the potential for improved productivity through breeding seems to overlook evidence that current yields are less than 50% of the yields shown to be technically feasible under farmers’ growing conditions (Scott *et al*., 2019a). In SSA, some observers have estimated potato production could be negatively impacted by over 14% in 2030 due to climate change (Jarvis *et al*., 2012). Others posit that to mitigate potential yield declines at lower elevations (<1000 m) due to rising temperatures in SSA will require specific crop adaptations such as switching to more heat‐tolerant potato cultivars (Raymundo *et al*., 2020). In LAC, the development of heat‐ and drought‐tolerant cultivars will also be needed to address climate change. In all regions, continued product diversification (e.g. tighter grading, more convenient packaging; in easy‐meal format; promotion of local varieties for niche markets) will help potato value chain participants serve increasingly demanding urban consumers.

Creative ways of reminding consumers of the importance of eating potatoes with their skins intact to capture their full nutritional benefits also merit attention. For example, the marketing of the smallest tubers as cocktail potatoes has converted them into a fashionable and nutritious side dish when served alongside meat, fish and other food items as part of a regular meal and increased their market value.

### Banana and plantain

Future prospects for dessert bananas are limited primarily by the sector’s capacity to address long pending issues related to the crop’s narrow genetic base and efforts to galvanise public–private partnerships to overcome it (Ortiz & Swennen, 2013; Brown *et al*., 2017; Lescot, 2018). Although plantain is overwhelmingly produced by the poorest, low‐income households in SSA, efforts to realise plantain’s potential have been hampered by pests, diseases and underfunded crop development programmes (Kwa & Temple, 2019). Additional key bottlenecks to address include research on improved post‐harvest management to reduce losses and more convenient ways to facilitate greater urban consumption.

### Sweetpotato

With the advent of climate change, sweetpotato cultivation may prove to be a valuable alternative to more input‐intensive, irrigation‐dependent commodities in the decades ahead. As research results related to a combination of improved varieties with virus resistance, post‐harvest and institutional innovations in East and Southern Africa demonstrate (Low & Thiele, 2020) the combination of local breeding programmes, consumer education about the nutritional benefits to be captured from eating provitamin A enriched, orange flesh sweetpotato and a commitment by national governments to allocate resources to that end offer an established road map for future activities. Similar integrated efforts might also be of benefit elsewhere in SSA as well as Asia including China (Li *et al*., 2018) and LAC so that sweetpotato’s full potential, neglected for decades, might be fully realised. In that regard, as many countries in Asia and Africa become more urbanised, the diffusion of information on sweetpotato’s reputation as a 'superfood' (i.e. as a particularly good source of vitamins, minerals and for certain varieties anti‐oxidants) might well facilitate that process (Woolfe, 1992).

Previous research has also mapped out the variety of processed products that can be made from sweetpotato including flour, feed and starch while acknowledging that such products are often unknown in regions such as SSA (Andrade *et al*., 2009). Combining that information with a prioritised assessment of the market potential for different processed products for human consumption would also help prioritise breeding initiatives and thereby reconcile the need for greater competitiveness with limited resources while starting late in the race to carve out market niches going forward.

### Yam

Key constraints to greater yam production include the fact that 30% of production goes for seed, the high labour costs (e.g. for making mounds, mulching, staking and harvesting by digging vertically), pests and diseases including at the post‐harvest stage as well as the traditional reluctance of yam producers to exchange and/or buy smaller yam tubers used for seed (Nweke, 2016). Addressing these constraints through improved seed systems and innovative approaches to facilitate greater grower adoption of such material is one key component of greater competitiveness (Sanginga & Mbabu, 2015). Similarly, creative ways to ensure greater adoption of better post‐harvest handling of yam could also facilitate both a reduction in losses from spoilage and weight loss and more effective use of output for food and income.

### All RTBs

Over the years, several authors have pointed to the need for better statistics on production and utilisation of RTBs, particularly in SSA to monitor food security and estimate impact among other things for these crops (Scott *et al*., 2013a, 2013b; Nweke, 2016; Spencer & Ezedinma, 2017; Lescot, 2018, 2020). RTBs do represent special challenges associated with monitoring indicators for these crops, although not unique in that regard. These would include continuous harvests during the year; on small, often non‐contiguous plots; for small farms often in combination with other crops, in isolated locations; and where what is harvested can be used for both consumption and as seed. Having said that, the persistent gaps, spikes and dramatic revisions in times‐series statistics along with data published on one RTB that subsequently is shown to be for another make improving the quality of these data an important part of future efforts to improve their potential development.

With growing urbanisation – particularly in SSA and Asia – and the emergence of pandemics such as the COVID‐19 combined with concerns about perishability that have been traditionally associated with RTBS and related processed products for human consumption, packaging and handling seem certain to assume even greater importance in the years ahead.

## Conclusions

In summary, this paper set out to provide review of RTBs in the agri‐food systems of developing countries via the analysis of FAO data and a select review of the literature. Based on that consolidated assessment, certain key developments stand out:

- the surge in production and use of cassava and yam in SSA and the growing importance of total output and utilisation of those commodities in that region;
- the boom for potato, cassava and banana production in Asia and a concentration of global potato and banana output and utilisation in that region;
- the major decline in output of sweet potato in China contrasting with its recent rise in output in East and Southern Africa;
- the increasing market orientation of RTBs heretofore considered subsistence crops in developing countries; for example, potato is predominately a cash crop in Asia as is cassava in SEA; plantain remains the exception;
- growing recognition of shifting tastes and preferences as well as use patterns for RTBs and their influence on production decisions and utilisation outcomes be they for food, feed or industrial uses now and in the years ahead;
- since 1988–90, growth rates for several RTBs higher than those for those for several cereal crops in Africa, Asia and LAC suggesting their growing importance in developing country agro‐food systems; and
- current trends suggest several RTBs either already have (yam) or will likely surpass (cassava and potato) estimates for their production in 2030.

These findings also point to the importance of greater recognition that RTBs have and will most likely play in addressing the food, nutrition and income needs of growing numbers of producers and consumers in emerging economies in the future. More specifically, these findings call strongly for renewed international and national support to carry out the research needed to address the constraints to production, utilisation and consumption for each of these commodities according to the regional crop and product‐specific priorities detailed above.

Strategies to promote greater public–private collaboration to enable RTBs realise their full potential also merit catalysing. These would include up‐grading cold storage capacity of potato in South Asia and re‐emphasis of consumption of potato with the skin intact across Asia, Africa and LAC. For cassava, the commercial potential of better processing for feed, starch and other industrial uses in SSA calls for coordinated efforts. Overcoming the narrow genetic base for dessert banana is a clear necessity. Helping to promote greater consumption of improved sweetpotato cultivars with high content of beta‐carotene is another opportunity as is enhancing the export potential of yam to markets outside of SSA as diets continue to evolve across the world. An added, emerging challenge is how to address the changing food requirements in growing urban areas in SSA currently frequently catered to by processed, often imported, cereal products.

## Author Contribution

**Gregory J Scott:** Conceptualization (lead); Data curation (lead); Formal analysis (lead); Methodology (lead); Writing‐original draft (lead); Writing‐review & editing (lead).

### Peer Review

The peer review history for this article is available at https://publons.com/publon/10.1111/ijfs.14778.

## Data Availability

All data presented in this study are based on the publicly available secondary sources cited and listed in the references. Ethics approval was not required for this paper as it analyses publicly available data and previously published documents.

## Notes

^1^ In the DRC, more cassava leaves are reportedly sold than the roots (T. Abdoulaye, personal communication).

^2^ With the breakup of the Soviet Union what was part of potato production in Europe – although actually taking place in Central Asia, became reclassified as such. For all these reasons then, from the early 1990s, Asia – no longer Europe – became the centre of global potato production (Scott & Suarez, 2012a).

^3^ IFPRI (2019) treats roots and tubers as a separate category. Banana and plantain are part of fruits and vegetables (F&V). F&V includes numerous commodities, thereby preventing meaningful comparisons between 2016–18 production for banana and plantain versus projections for F&V for 2030 and 2050. See IFPRI (2019) for additional methodological details.

^4^ Previous projections for RTBs estimated that they would reach 1400 million t by 2050 (Rosegrant *et al.,* 2017 as cited by Petsakos *et al*.*,* 2019). Based on 2016–18 output levels (Table 4) that total could be achieved by an annual average growth rate of just over 1.5%.
